# Molecular interplay between glycogen synthase kinase 3 beta and A-kinase anchoring protein 11 in bipolar disorder: a narrative review

**DOI:** 10.3389/fnmol.2026.1813800

**Published:** 2026-06-12

**Authors:** A Ashvil, Prakash Patil, Shrinivasa Undaru Bhat, Uday Venkat Mateti, Allen Pinto, Deepak C Gopinath, Juno Jerold Joel

**Affiliations:** 1 Department of Pharmacy Practice, NGSM Institute of Pharmaceutical Sciences (NGSMIPS), Nitte (Deemed to be University), Mangalore, Karnataka, India; 2 Central Research Laboratory, KS Hegde Medical Academy (KSHEMA), Nitte (Deemed to be University), Mangalore, Karnataka, India; 3 Department of Psychiatry, KS Hegde Medical Academy (KSHEMA), Justice KS Hedge Charitable Hospital (JKSHCH), Nitte (Deemed to be University), Mangalore, Karnataka, India

**Keywords:** AKAP11, edgetic mutation, genetic association, GSK3β, lithium therapy, scaffolding proteins

## Abstract

Bipolar disorder (BD) is a complicated psychiatric condition which is determined by episodic mood instability, yet its underlying biological foundation still remains poorly understood. A-kinase anchoring protein 11(AKAP11) has been identified a high-confidence risk gene through recent large scale genomic investigations, its ultra-rare protein truncating variants lead to seven fold increased risk for BD and Schizophrenia. This narrative review aims to examine the molecular interplay between AKAP11, a multivalent scaffolding protein, and Glycogen synthase kinase-3β (GSK3β), a crucial regulator of synaptic plasticity and the primarily suspected target of lithium therapy. AKAP11 acts as a structural chassis, which sequesters GSK3β amongst discrete subcellular microdomains to facilitate its localised suppression by PKA-mediated phosphorylation. We focus how this protein–protein interface is selectively disrupted by “edgetic” mutations, which leads to escape of GSK3β from homeostatic control through spatial mislocalization. The resultant cellular abnormalities consist of impaired dendritic spine stability, proteostatic stress caused by defective autophagy of signalling complexes, and reduced synaptic transmission. Changes in excitatory and inhibitory balance and signalling stability, that are linked to bipolar disorder, may be facilitated by these pathways. However, there are limited evidences stating that direct disruption of AKAP11-GSK3β interaction may lead to episodic-mood state transition. Therefore, even though the clinical significance of AKAP11 and GSK3β interaction is yet to be established, its further investigation is a potential therapeutic target.

## 1 Introduction

The state of biological psychiatry today represents a paradigm shift. It is moving from the constraints of the monoamine hypothesis to the multidimensional systems model of psychiatry (Tanaka, 2025). Historically, the most popular view indicated that bipolar disorder is primarily driven by dysregulations within serotonin, norepinephrine, or dopamine pathways (Hirschfeld, 2000; Schildkraut, 1965). Although this paradigm of illness contributed to the development of pharmacotherapies for a variety of diseases, it often overlooked the latency of illness onset, the prevalence of treatment-resistant illness, and the complex episodic pattern of many of these diseases (Hirschfeld, 2000). A recent study has indicated that these diseases are perhaps more usefully considered as disorders of spatiotemporally regulated signalling, characterized by disrupted intracellular signalling cascades and impaired synaptic plasticity (Jiang et al., 2022). In this context, the spatial organization of kinases and phosphatases is critical in determining the specificity of signal transduction (Imiruaye et al., 2025; Kandilakis and Papatheodoropoulos, 2025). Emerging evidence suggests that the interaction between GSK3β and AKAP11 may play a role in intracellular signaling processes relevant to mood regulation; however, its direct contribution to mood modulation in humans remains unclear (Palmer et al., 2022; Whiting et al., 2015).

Glycogen synthase kinase-3β is a highly conserved serine/threonine kinase that is constitutively active at basal conditions. It is responsible for activating and regulating glycogen synthase through inhibitory processes, including phosphorylation of Serine 9 (Ser9) at site 8 (Ombrato et al., 2015). GSK3β regulates neurogenesis, microtubule stability, and circadian rhythms in the central nervous system (Forde and Dale, 2007; Beurel et al., 2015). The pathophysiology of bipolar disorder is consistently associated with hyperactivity or mislocalization of the kinase, particularly through its effect on synaptic architecture (Jope et al., 2017). GSK3β has been shown to induce the erosion of dendritic spine density as well as synaptic destabilization in NMDA receptor-dependent long-term depression (LTD) (Peineau et al., 2007). This enzymatic hub is of profound pharmacological interest, as it remains the primary hypothesized target of lithium, the gold-standard treatment for bipolar disorder (Peineau et al., 2007; Ochs et al., 2015; Klein and Melton, 1996).

The most compelling genetic evidence comes from two large-scale rare variant sequencing efforts. The Bipolar Exome consortium analyzed whole-exome sequencing data from 13,933 individuals with bipolar disorder and 14,422 matched controls, identifying a significant excess of ultra-rare protein-truncating variants in evolutionarily constrained genes. When these findings were combined with data from the Schizophrenia Exome Meta-Analysis, which included 24,248 cases and 97,322 controls, AKAP11 emerged as the sole exome-wide significant risk gene shared across both disorders, with an odds ratio of 7.06 and *p* = 2.83 × 10^−9^—among the largest effect sizes reported in psychiatric genetics to date (Palmer et al., 2022). Critically, this genetic signal is driven by ultra-rare protein-truncating variants present in a very small fraction of bipolar disorder cases, a point with direct implications for how therapeutic strategies based on this finding should be interpreted, as discussed in Section 8. AKAP11 functions as a multivalent scaffolding protein, organizing PKA, PP1, and GSK3β into discrete subcellular microdomains and coordinating multiple signalling pathways (Segura-Roman et al., 2025).

The direct molecular interaction between GSK3β and AKAP11 is a sophisticated, phosphorylation-dependent process. Previous research indicates that active GSK3β phosphorylates AKAP11 at Threonine 1,132 (Thr1132), a modification that initiates and stabilizes the recruitment of the kinase into the enzyme scaffold (Whiting et al., 2015). This recruitment allows for the precise, localized inhibition of GSK3β by anchored PKA, which phosphorylates the kinase at Ser9 to trigger its release from the complex. Disruption of this interaction through truncating mutations affecting the protein–protein interface may alter the spatial regulation of signaling complexes and has been associated with changes in synaptic function in experimental systems (Palmer et al., 2022; Whiting et al., 2015; Lee et al., 2024).

Moreover, recent studies in 2025 have found AKAP11 as a particular autophagy receptor of the PKA-RI complex, aside from its role in scaffolding. Deletion of AKAP11 leads to an improper accumulation of PKA regulatory subunits and changes in GSK3β activity, bringing into play the relationship between proteostasis and the sudden shift in mood seen in bipolar disorder (Lee et al., 2024). This review focuses on the molecular interrelationship of AKAP11 with GSK3β, an issue that is not elaborated within bipolar disorder. While new data point to their interaction, there is still a serious gap in how the disruption of this complex contributes to psychiatric disease. The interaction between AKAP11 and GSK3β is fascinating not only from a molecular viewpoint but also from a very important pharmacological perspective, especially in targeted therapy of bipolar disorder.

## 2 Glycogen synthase kinase-3β: molecular and functional background

**Figure 1:**
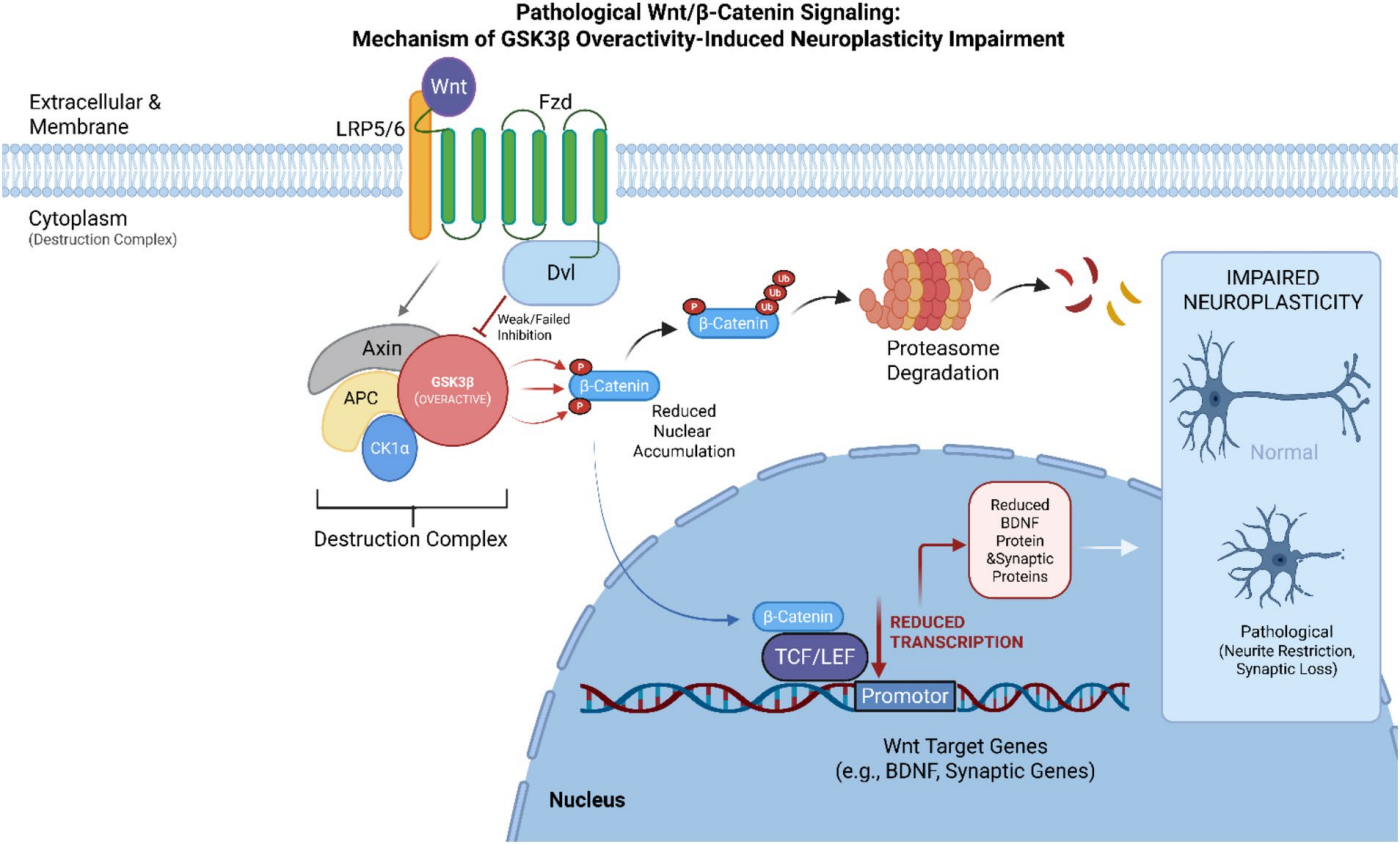
This graphic depicts Glycogen synthase kinase-3β overactivity in the Wnt signalling pathway, leading to neurite retraction.

A crucial regulator of brain signalling, GSK3β integrates numerous intracellular cascades, including Wnt (Jope et al., 2017), insulin (Lee and Kim, 2007), inflammation, and cellular stress (Jope et al., 2017). Originally characterized for its role in glycogen metabolism (Lee and Kim, 2007), GSK3β is now recognized as a critical determinant of neuronal fate (Liu et al., 2005), structural plasticity (Cymerman et al., 2015), and homeostatic mood regulation (Li and Jope, 2010), (Figure 1).

### 2.1 Constitutive activity and biochemical uniqueness

Unlike most signal-transduction kinases, GSK3β has strong constitutive activity even in the absence of an activating input. This always-on position indicates that cellular signalling through this kinase is predominantly regulated by inhibitory mechanisms rather than activation triggers. This unique biochemical status positions GSK3β as a persistent molecular brake or facilitator, depending on the specific subcellular compartment and the presence of inhibitory phosphorylation (Dajani et al., 2001).

GSK3β is considered one of the most atypical kinases in the human genome due to several distinctive regulatory features. First, efficient phosphorylation of most GSK3β substrates requires prior “priming” phosphorylation by a separate kinase at serine or threonine residues located four to five amino acids C-terminal to the GSK3β target site. Second, unlike the majority of signal-transduction kinases, GSK3β exhibits constitutive catalytic activity under basal cellular conditions. Third, phosphorylation of GSK3β at an N-terminal serine residue results in inhibition of its kinase activity. This inhibitory phosphorylation is mediated by members of the AGC kinase family, such as Akt, and typically occurs downstream of growth factor–dependent phosphatidylinositol 3-kinase (PI3K) signalling pathways (Kennelly and Krebs, 1991; Cross et al., 1995; Sutherland et al., 1993).

**Figure 2:**
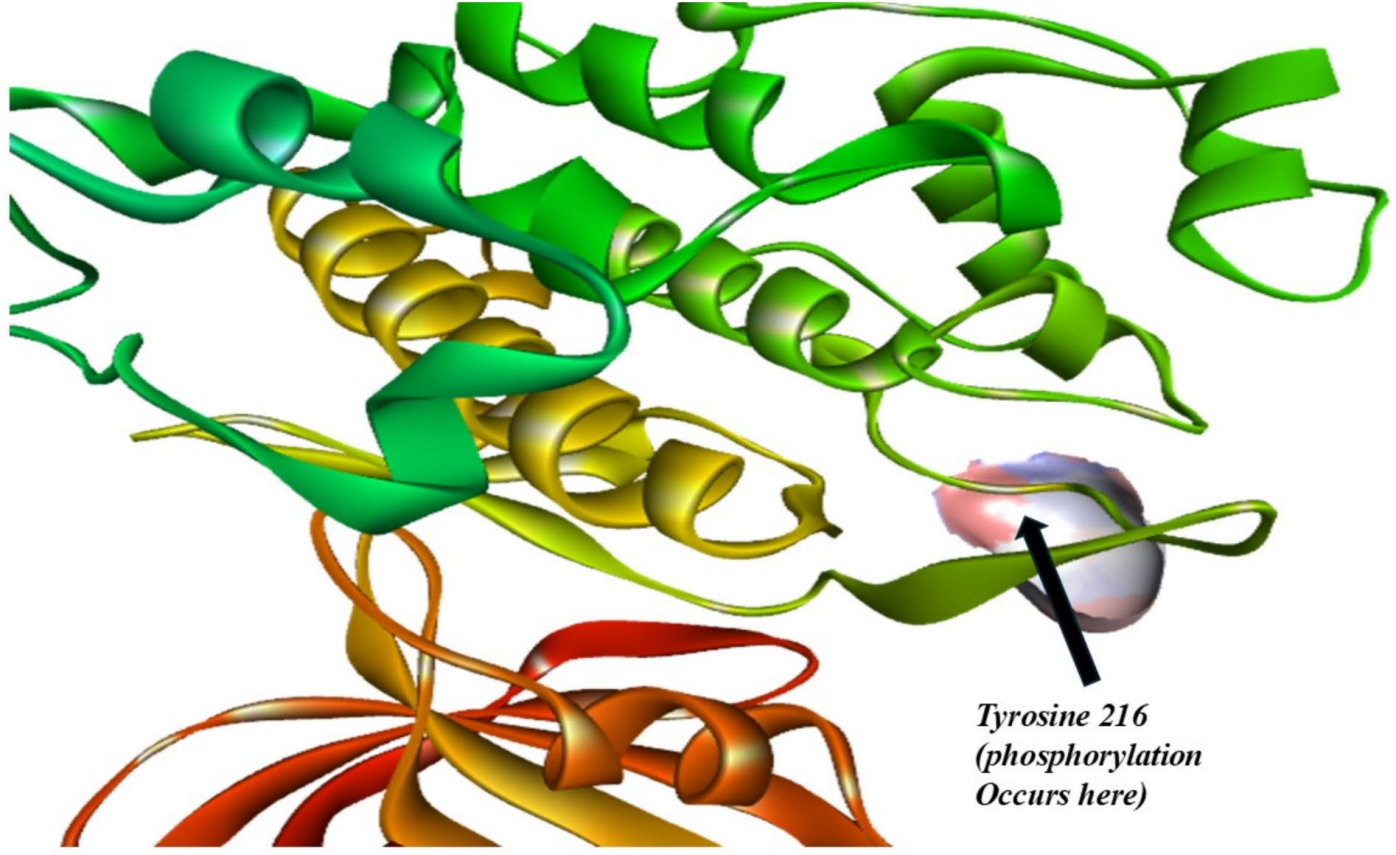
The 3D structure of Glycogen synthase kinase-3β showing the Tyr 216, where phosphorylation occurs to retain its full activity (PDB ID: 1H8F).

Full catalytic efficiency of the enzyme typically requires phosphorylation of Tyrosine 216 (Tyr216) within the activation loop, a modification that often occurs co-translationally during the enzyme’s maturation (Cole, 2013). However, the most vital regulatory checkpoint during the enzyme’s lifespan is the inhibitory phosphorylation of the Serine 9 (Ser9) residue. When Ser9 is phosphorylated, typically by upstream kinases such as AKT (Protein Kinase B) or Protein Kinase A (PKA), the amino-terminal tail of GSK3β undergoes a conformational shift. This phosphorylated tail acts as a pseudo-substrate, binding to the enzyme’s own positively charged pocket and physically occluding the entry of exogenous primed substrates (Dajani et al., 2001), (Figure 2).

### 2.2 Regulation of synaptic plasticity and architecture

In the central nervous system, GSK3β is a fundamental modulator of synaptic strength and structural integrity. It is specifically implicated in the induction of N-methyl-D-aspartate receptor (NMDAR)-dependent long-term depression (LTD), a physiological process characterized by the weakening of synaptic connections. During LTD, active GSK3β phosphorylates postsynaptic density protein 95 (PSD95) and kinesin light chain 2, which facilitates the internalization of alpha-amino-3-hydroxy-5-methyl-4-isoxazolepropionic acid (AMPA) receptors from the postsynaptic membrane (Jaworski et al., 2019; Aceto et al., 2019; Nelson et al., 2013).

**Figure 3:**
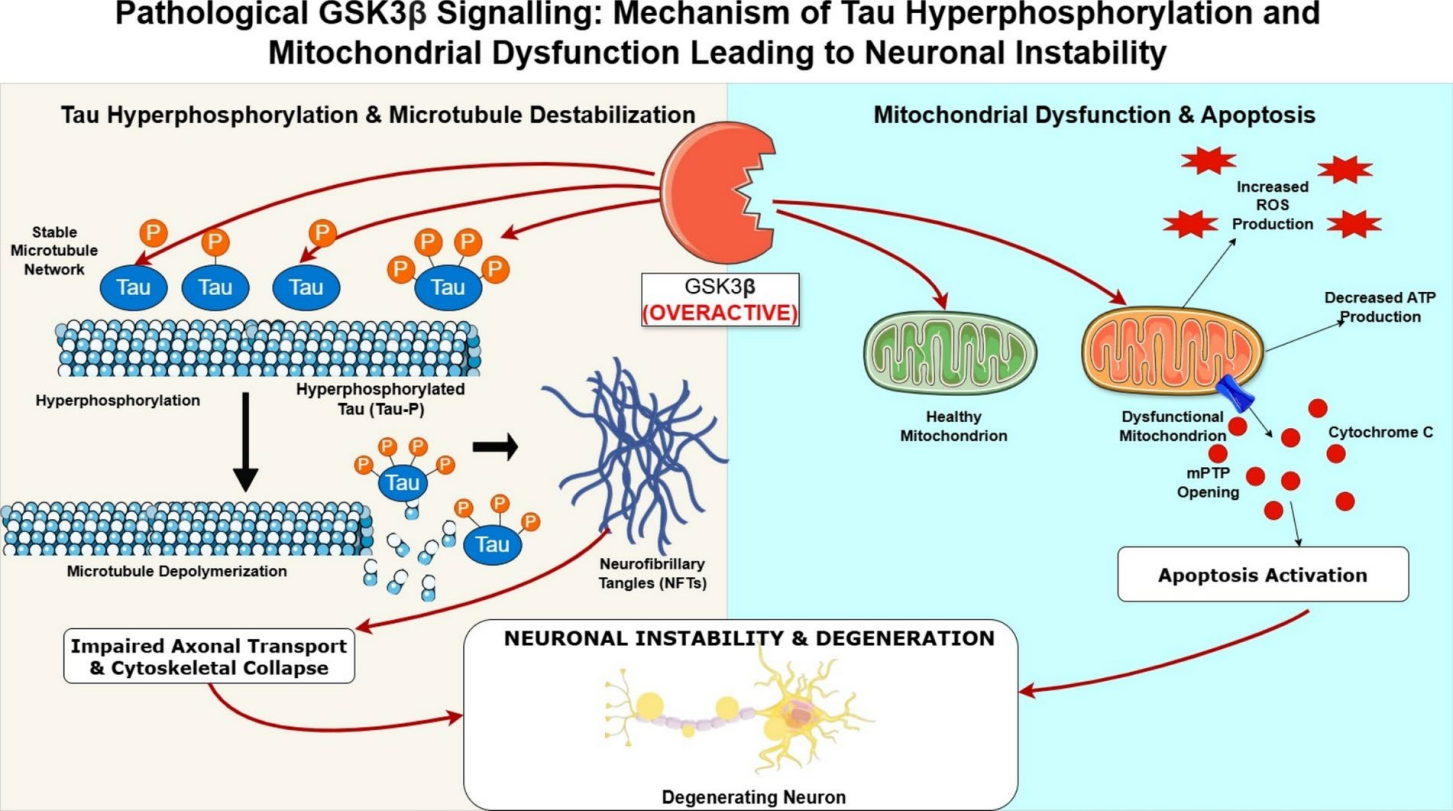
Aberrant activation of glycogen synthase kinase-3β leads to neuronal instability and degeneration of neurons. licensed under Attribution 4.0 International, CC by 4.0. (https://creativecommons.org/licenses/by/4.0/).

The kinase further exerts control over the physical architecture of the neuron through its interactions with the cytoskeleton. GSK3β phosphorylates various microtubule-associated proteins, such as Tau (Medina et al., 2011) and MAP1B (Goold and Gordon-Weeks, 2005), thereby regulating microtubule stability and neurite outgrowth. According to experimental data, dendritic spine stability depends on GSK3β activity being kept within a tight homeostatic range. The total loss of neuronal GSK3β also decreases spine density and attenuates excitatory transmission, frequently through the abnormal buildup of beta-catenin, whereas overactivity is typically associated with synaptic degradation. In BD, mitochondrial dysfunction is also caused by GSK3β overactivity (Giménez-Palomo et al., 2024), (Figure 3).

### 2.3 Circadian rhythms and neurogenesis

GSK3β is an essential part of the molecular clockwork that extends beyond synaptic dynamics. By phosphorylating crucial clock proteins like Period 2 (PER2) and modifying the nuclear translocation of Rev-Erb alpha, it controls circadian rhythms (Wu et al., 2008). This temporal regulation is especially important for bipolar disorder, which are usually characterized by significant disruptions in sleep–wake cycles and diurnal energy rhythms. GSK3β inhibits the canonical Wnt signalling pathway by phosphorylating beta-catenin, causing ubiquitination and proteasomal degradation. This limits the proliferation and differentiation of neural progenitor cells (Figure 1), (Muneer, 2017).

### 2.4 Pathophysiological link to mood instability

Bipolar disorder is characterized by dysregulation of GSK3β activity (Inkster et al., 2018). Elevated kinase activity has been identified in many brain regions during depressive and manic states, and its capacity to influence the transition between these states is of particular therapeutic importance (Jacoby et al., 2016). Lithium is a mood stabilizer used in bipolar disorder; its major target is GSK3β. The drug exerts its therapeutic effects at two levels: by directly inhibiting the kinase through competition with magnesium ions at the catalytic site and indirectly enhancing inhibitory Ser9 phosphorylation (Chatterjee and Beaulieu, 2022).

**Figure 4:**
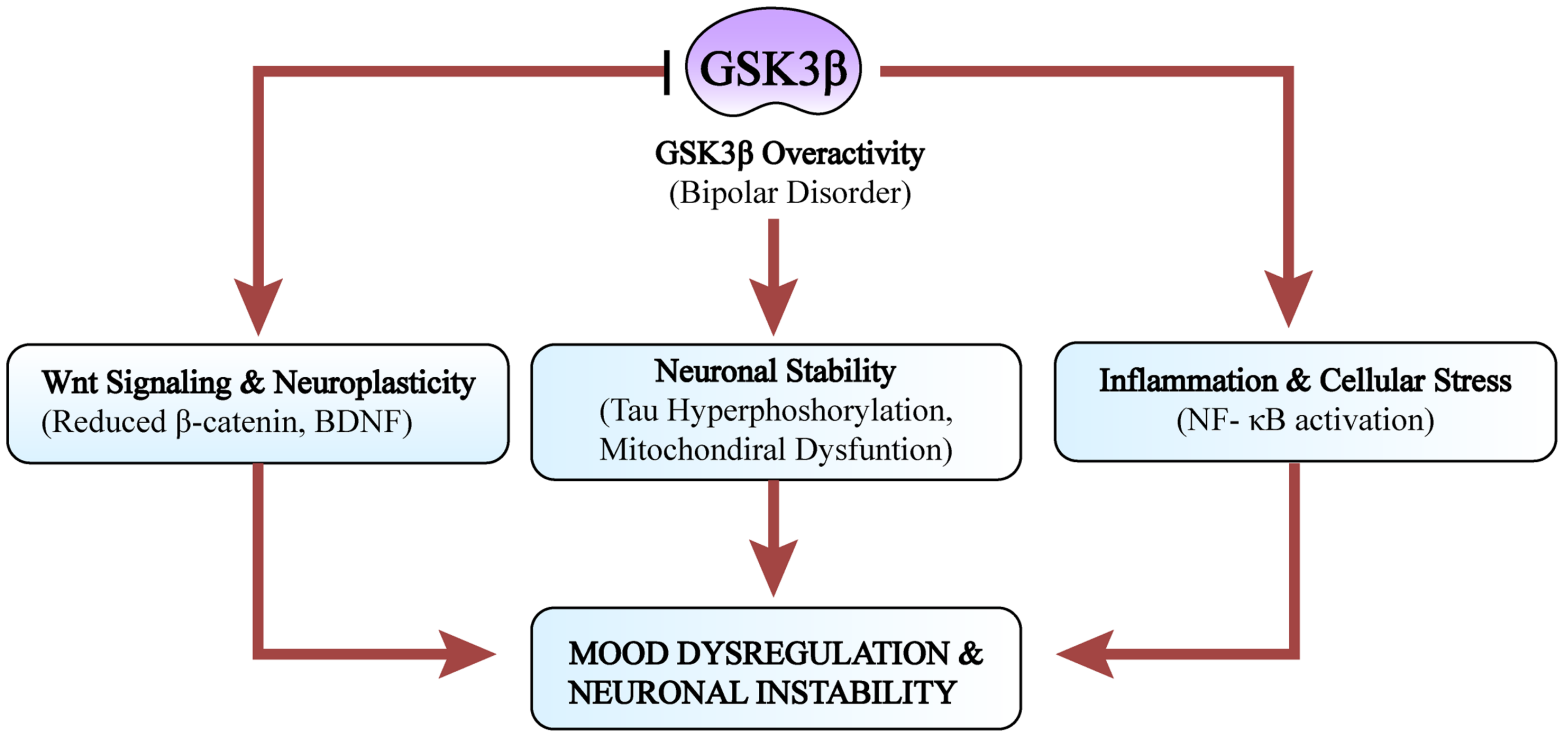
The above graphic shows how glycogen synthase kinase-3β overactivity, which is linked to bipolar disorder, causes mood dysregulation by interfering with Wnt signalling, increasing inflammation, and disrupting neural stability. In particular, this pathway demonstrates how NF-κB activation, mitochondrial malfunction, and Tau hyperphosphorylation all lead to neuronal instability.

GSK3β is a promiscuous kinase with hundreds of possible substrates, requiring precise spatial control for signalling specificity (Song et al., 2025). Neurons attain precision by recruiting GSK3β to specific scaffolding proteins (Kim et al., 2011). Without these interaction partners, the kinase would lack the appropriate spatial context, resulting in abnormal phosphorylation of non-target proteins and contributing to signalling instability in psychosis and bipolar disorder. The discovery of AKAP11 as a high-affinity scaffold for GSK3β is a significant step toward understanding how this enzymatic hub is linked to certain neural activities (Whiting et al., 2015), (Figure 4).

## 3 A-kinase anchoring protein 11: structural and regulatory role

A-kinase anchoring protein 11 (AKAP11) is a big, multivalent scaffolding protein with 1,901 amino acids (see UniProt Primary Accession number Q9UKA4). As a major member of the AKAP family, its principal physiological function is to coordinate the spatial and temporal distribution of intracellular signalling complexes. By providing a structural chassis for many enzymes, AKAP11 guarantees that signal transduction is a confined and precise biochemical process rather than a widespread, random event (Whiting et al., 2015). A recent study has elucidated the mechanism underlying the impact of AKAP11 deficiency on human neurons. By employing iPSC-derived neuronal cells, it was found that loss-of-function mutations in AKAP11 resulted in global transcriptional and epigenetic dysregulation in terms of DNA methylation and histone acetylation profiles. Specifically, an unusual enhancement in intergenic and intronic enhancer activation that contained PBX2 and NF1 regulatory elements was linked to aberrant expression of genes encoding proteins responsible for transcriptional activity, cytoskeletal structure, and cytokines. Simultaneously, the ribosomal pathway was significantly repressed, which is analogous to observations made on postmortem brain samples from patients with bipolar disorder and schizophrenia and synaptic proteomics in AKAP11-mutant mice (Farhangdoost et al., 2025).

### 3.1 Architectural scaffolding and multimodal anchoring

AKAP11 acts as a molecular hub, connecting a wide range of signalling effectors into distinct subcellular microdomains (Whiting et al., 2015). Its principal interaction is binding to the regulatory subunits (RI and RII) of Protein Kinase A (PKA) via highly conserved amphipathic alpha-helices (Falcone et al., 2025). AKAP11 holds both PKA and PP1 in the same place. This permits the cell to quickly turn up or down protein activity exactly where it’s needed, keeping signalling balanced and precise (Segura-Roman et al., 2025).

In addition to kinases and phosphatases, the AKAP11 interactome includes:

*IQGAP1*: A regulator of actin cytoskeleton and microtubule dynamics (Song et al., 2025).
*VAPA/VAPB*: ER-resident proteins that facilitate the recruitment of AKAP11 to the endoplasmic reticulum (Liu et al., 2025).
*GABAC receptors*: Suggesting a role in the spatial modulation of inhibitory neurotransmission (Liu et al., 2025).

### 3.2 Precision signalling and proteostatic control

AKAP11 expression is significantly elevated in regions of the central nervous system that are essential for affective and cognitive processing, such as the striatum, hippocampus, and prefrontal cortex (Song et al., 2025). The protein is responsible for the integrity of the signal transduction event by acting to localize enzymes to the site of their target, either at the synapse or the cytoskeleton. Localization of the target is essential for the event of neurotransmission, and this is achieved by AKAP11, which localizes the PKA-GSK3β complex to carry out the phosphorylation-mediated inhibition of the target.

In 2025, a groundbreaking study redefined AKAP11 as a particular autophagy receptor instead of a static scaffold. AKAP11 interacts with VAP proteins at the ER via its functional FFAT motif, facilitating the autophagic turnover of the PKA-RI complex. This proteostatic activity is necessary for maintaining the proper ratio of PKA subunits at the synapse (Lee et al., 2024). In the absence of functioning AKAP11, PKA-RI complexes accumulate pathologically, causing activity distortion in which basal kinase activity is increased while signal-responsive dynamics are suppressed.

### 3.3 Direct association of A-kinase anchoring protein 11 with glycogen synthase kinase-3β

AKAP11 interacts with GSK3β via a specific docking area, similar to Axin-mediated GSK3β binding in Wnt signalling. Evidence suggests that phosphorylating AKAP11 at Thr1132 by active GSK3β may improve the stability of this connection, indicating a potential feedback mechanism related to mood-associated signalling pathway (Whiting et al., 2015).

This direct physical link serves as a primary mechanism for the localized inhibition of GSK3β. Within the AKAP11 microdomain, anchored PKA is positioned to phosphorylate GSK3β at the inhibitory Serine 9 (Ser9) site, thereby terminating its localized signalling activity. The breakdown of this interaction in carriers of rare protein-truncating variants has been associated with altered spatial regulation of GSK3β in experimental models, including changes in synaptic localization observed in cell-based systems (Whiting et al., 2015). Whether these molecular alterations translate to the signalling instability and synaptic changes implicated in bipolar disorder remains an open question that has not been directly tested in human tissue or clinical cohorts.

## 4 Molecular interaction between glycogen synthase kinase-3β and A-kinase anchoring protein 11

The physical connection between AKAP11 and GSK3β forms a fundamental regulatory axis that distinguishes the kinase’s specialized signalling pool from its more general metabolic and developmental functions. Through a multi-step biochemical process, this interaction is a dynamic, signal-responsive assembly that controls the spatial accuracy of neuronal cascades rather than a static meeting (Whiting et al., 2015; Wang et al., 2022).

Before getting into the molecular details of how AKAP11 and GSK3β interact, it helps to first understand how disease-associated variants are thought to break this interaction. Genetic studies have identified two main categories of pathogenic AKAP11 variants. The first category includes protein-truncating variants, such as nonsense and frameshift mutations, which destroy the scaffold protein entirely. The second category includes missense variants that work through what is called an edgetic mechanism. Rather than eliminating the whole protein, these variants damage only one specific binding interface while leaving the rest of the scaffold structurally intact and functional for its other partners (Cui et al., 2023; Sahni et al., 2013).

**Figure 5:**
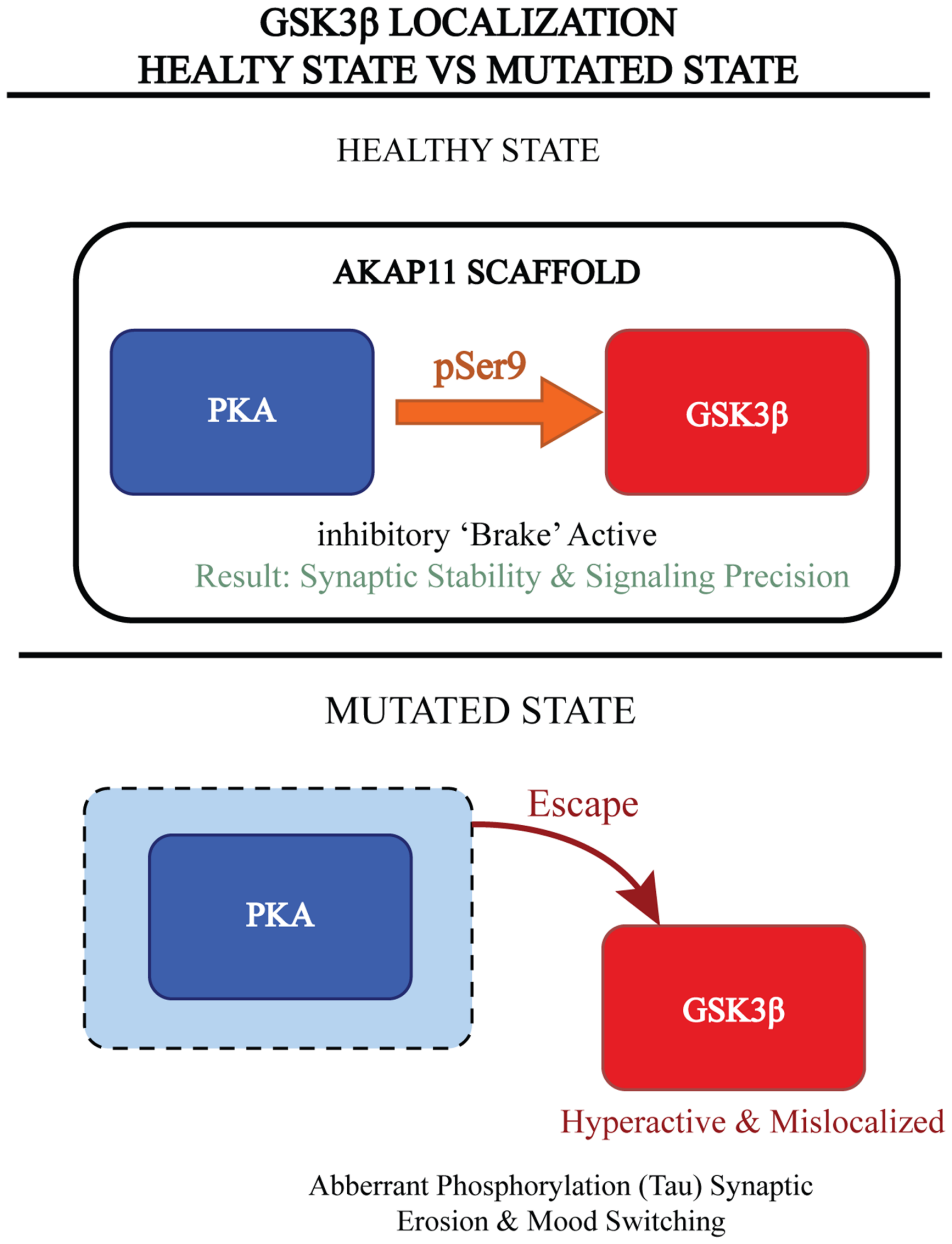
Glycogen synthase kinase-3β localization.

To understand why this distinction matters, it helps to think of the AKAP11 protein as a hub with multiple connections. Classical loss-of-function mutations remove the entire hub from the network. Edgetic variants, by contrast, cut only one specific connection while leaving all the others in place. In the case of AKAP11, variants that cluster within the GSK3β interaction domain specifically block GSK3β from being recruited to the scaffold, while the scaffold continues to bind PKA regulatory subunits, PP1, and other partners normally (Figure 5).

### 4.1 Auto-recruitment mechanism and binding domains

The GSK3β–AKAP11 interaction is governed by an activity-dependent recruitment mechanism centered on Thr1132 within the 1,009–1,305 amino acid region of AKAP11. Active GSK3β phosphorylates AKAP11 at this residue, stabilizing its own docking within the scaffold, a positive feedback loop confirmed by peptide array and *in vitro* phosphorylation experiments showing that phosphorylation at Thr1132, but not the adjacent Thr1128, is required for binding. Cellular validation in HEK293 cells using the T1132A mutation eliminates this association entirely, establishing Thr1132 as the primary recruitment trigger. Constitutively active GSK3β variants show enhanced AKAP11 binding, further supporting a phosphorylation-gated assembly model (Whiting et al., 2015).

The primary phosphorylation site mediating the interaction between GSK3β and the 1,009–1,305 amino acid region of AKAP11 has been shown to be Thr-1132. GSK3β association requires phosphorylation at Thr-1132 but not Thr-1128, according to peptide array and *in vitro* phosphorylation experiments. The T1132A mutation eliminates binding, according to cellular validation in HEK293 cells, suggesting that Thr-1132 is crucial for the AKAP11–GSK3β interaction (Whiting et al., 2015).

AKAP11 functions as the definitive spatial regulator of GSK3β, ensuring its subcellular anchoring within critical neuronal compartments, particularly the synapses and membrane ruffles. This spatial positioning is the primary determinant of substrate accessibility; by sequestering the kinase within a microdomain, the scaffold brings it into immediate proximity with specific targets such as β-catenin and cytoskeletal regulatory proteins. In AKAP11-deficient neurons, there is a total failure to enrich GSK3*β* at the synaptic ruffle edge, demonstrating that the scaffold is structurally required to organize signalling during lamellipodia and synaptic formation (Whiting et al., 2015).

Anchored GSK3β exhibit context-dependent phosphorylation, where the kinase activity is restricted to a localized pool of proteins. This prevents the “promiscuous” kinase from aberrantly phosphorylating non-target substrates in the broader cytosol. The proximity of PKA and GSK3β on the same scaffold allows for immediate cross-talk: when local cAMP levels rise, anchored PKA is activated and can specifically phosphorylate the sequestered GSK3β at its Ser9 site, triggering its dissociation and terminating the signal (Whiting et al., 2015), (Table 1).

**Table 1:** AKAP11 coordinates kinase binding, activation, and localization at membrane ruffles through specific phosphorylation sites and structural motifs.

Interaction component	Molecular determinant	Physiological function	References
Recruitment trigger	Thr1132 phosphorylation	Initiates signal-responsive assembly of the AKAP11–GSK3 complex	Figures 1F–H, (Whiting et al., 2015)
Binding domain	GID (axin-homology)	Mediates the specific anchoring of GSK3 to AKAP11	Figures 1B,C, (Whiting et al., 2015)
Interface stability	FLL Motif (AKAP11)	Maintains proper orientation and stable interaction of GSK3 within the complex	Figure 1H, (Whiting et al., 2015)
Signal resolution	Ser9 phosphorylation (PKA)	Kinase inhibition by phosphorylation; triggers complex disassembly	Figure 1G, (Whiting et al., 2015)
Subcellular Hub	Membrane ruffles/synapses	Positions GSK3 near substrates such as β-catenin for localized signalling	Figures 2A,F–I, (Whiting et al., 2015)

### 4.2 Functional annotation and gene ontology (GO) enrichment of the A-kinase anchoring protein 11 with the glycogen synthase kinase-3β interactome

#### 4.2.1 GO enrichment analysis

**Figure 6:**
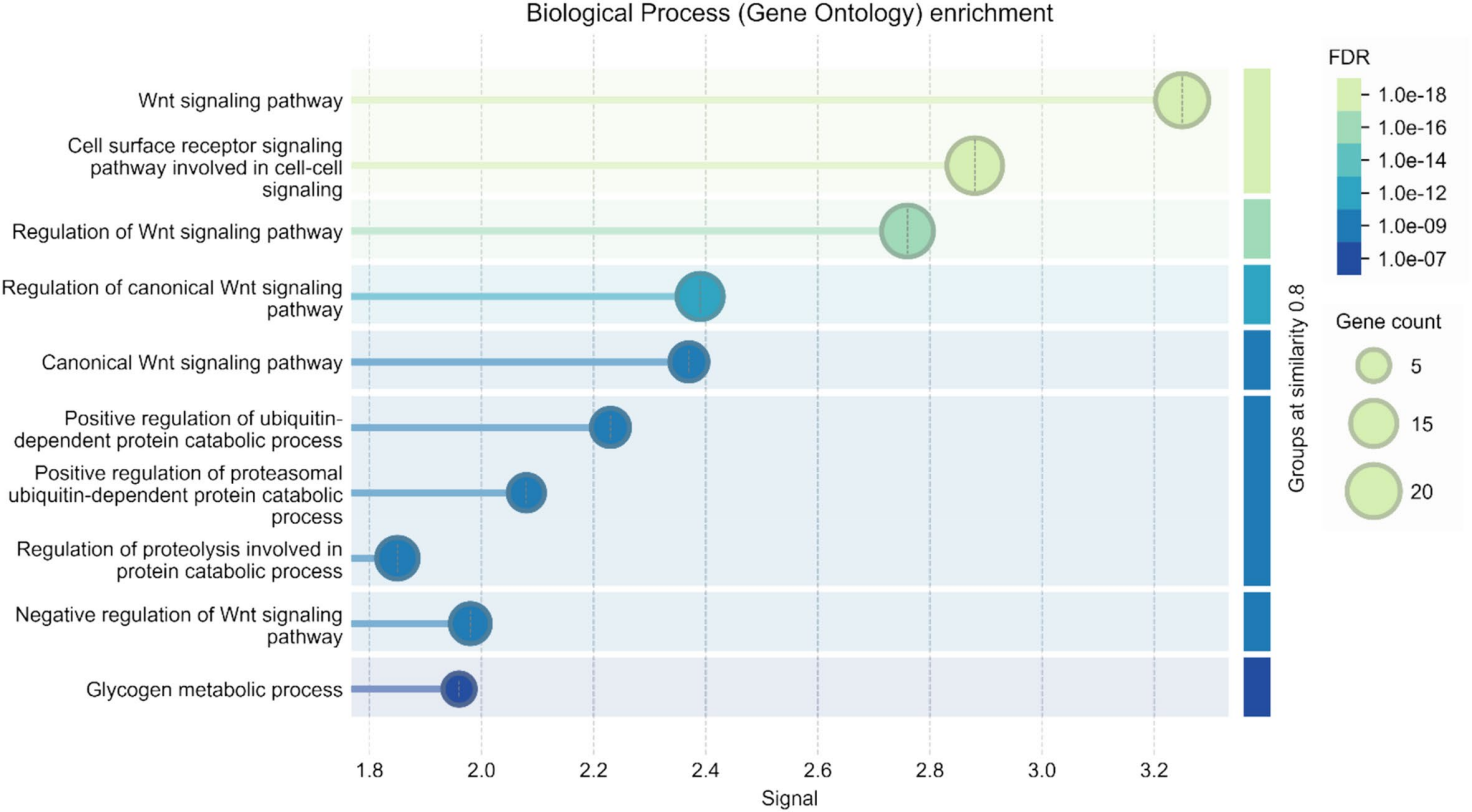
Comprehensive Gene Ontology (GO) enrichment analysis covering biological processes, molecular functions, and cellular components. The Wnt signalling pathway emerged as the most significantly impacted biological process, displaying the highest enrichment scores and statistical significance.

Gene Ontology enrichment analysis was performed using the STRING (Szklarczyk et al., 2025) database (v12.0) on the 50 manually curated brain-expressed proteins fetched from different protein databases constituting the AKAP11–GSK3β interactome. The full *Homo sapiens* proteome was used as the background reference set. Enrichment significance was assessed using the hypergeometric test with false discovery rate (FDR) correction applied using the Benjamini–Hochberg method; terms with FDR < 0.05 were considered statistically significant. Biological process, molecular function, and cellular component ontologies were all queried. The Wnt signalling pathway returned the highest enrichment score among biological processes, and ubiquitin-dependent proteolysis terms showed significant enrichment consistent with AKAP11’s established role as a selective autophagy receptor. The intersection of metabolic and signaling pathways suggests that disruption of AKAP11 may affect the regulation of localized signaling activity and proteasomal processes involving GSK3β. However, the extent to which these alterations contribute to bipolar disorder pathophysiology remains to be established (Figure 6).

#### 4.2.2 STRING PPI network

**Figure 7:**
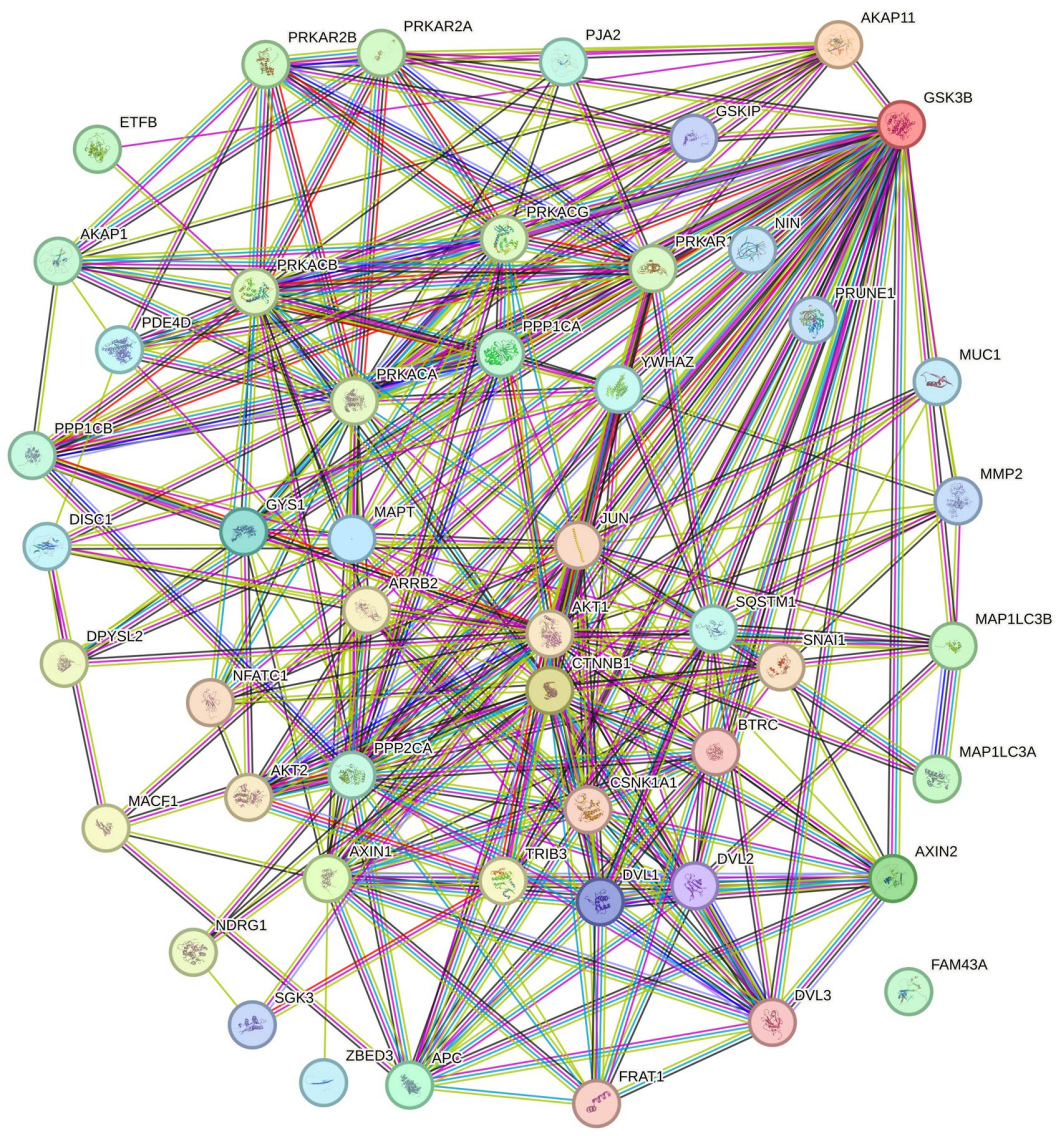
Protein–protein interaction network of 50 brain-related proteins. The statistically significant interaction between AKAP11 and GSK3β is clearly represented, confirming the presence of the AKAP11–GSK3β complex in humans.

The protein–protein interaction network was constructed using the STRING database (v12.0) (Szklarczyk et al., 2025) with a minimum required interaction score of 0.700 (high confidence). Network analysis was restricted to experimentally validated and database-curated interactions; text-mining channels were excluded to reduce false positives. The 50 input proteins were selected based on documented functional or physical association with GSK3β, PKA/AKAP scaffolding, Wnt/β-catenin signalling, cytoskeletal regulation, or neuronal survival pathways, as reported in peer-reviewed neuropsychiatric literature. Network topology metrics, including average node degree, local clustering coefficient, and PPI enrichment *p*-value, were computed within the STRING environment (Figure 7).

### 4.3 Pathogenic mislocalization and signalling instability

Edgetic variations in patients with schizophrenia and bipolar illness highlight the clinical significance of this relationship. These missense variants specifically occur within the GID or near Thr1132, selectively breaking the interaction interface while keeping the scaffold intact, in contrast to overall loss-of-function mutations that degrade the protein (Palmer et al., 2022).

GSK3β thus becomes mislocalized and escapes anchoring PKA’s temporal control. This results in signalling instability because aberrant phosphorylation of synaptic targets is driven by varying kinase activity. This mislocalization is linked to decreased dendritic spine density, particularly thin spines, and destabilization of the postsynaptic density in human iPSC-derived neurons and AKAP11 mutant mice (Farhangdoost, 2025).

## 5 Genetic evidence supporting the interaction of A-kinase anchoring protein 11 with of glycogen synthase kinase-3β

An important development in psychiatric genetics is the discovery of AKAP11 as a high-confidence risk gene for bipolar disorder, which connects uncommon, large-effect mutations to a specific intracellular signalling scaffold. Ultra-rare protein-truncating variations in AKAP11 have significantly greater impact sizes, allowing for clearer biological interpretation, in contrast to common variants found by genome-wide association studies, which individually impose little risk. These results support the theory that disruption of localized signalling microdomains contributes to the pathophysiology of bipolar disorder and schizophrenia, given AKAP11’s role in organizing PKA–GSK3β signalling complexes. However, the exact mechanistic implications are still unclear (Palmer et al., 2022).

### 5.1 Definitive risk in the BipEx and SCHEMA cohorts

Large-scale rare variant sequencing efforts provide the strongest genomic support. Whole-exome sequencing data from 13,933 people with bipolar disorder and 14,422 matched controls were analyzed by the Bipolar Exome (BipEx) consortium. The results showed a high enrichment of ultra-rare protein-truncating mutations in affected individuals. This burden, which indicates intolerance to loss-of-function variation, was most noticeable in genes under significant evolutionary pressure. When these results were combined with those from the Schizophrenia Exome Meta-Analysis (SCHEMA) collaboration, AKAP11 repeatedly showed up as a risk gene, with high statistical support and a substantial impact size (Palmer et al., 2022).

Current evidence indicates that rare deleterious variants in AKAP11 are preferentially enriched in psychosis-spectrum disorders, particularly bipolar disorder and schizophrenia (Lee et al., 2024; Thorgeirsson et al., 2025). Large-scale rare variant studies have not consistently reported enrichment of protein-truncating variants across several neurodevelopmental conditions, including autism spectrum disorder, epilepsy, and intellectual disability. This pattern suggests that AKAP11 disruption is more strongly associated with affective and psychotic disorders rather than broad neurodevelopmental impairment (Song et al., 2025; Farhangdoost et al., 2025). Emerging population-specific sequencing efforts in East Asian cohorts have identified additional rare coding variants in AKAP11 among bipolar disorder cases, suggesting that risk-associated variation may not be confined to European populations (Zhang et al., 2025). However, these findings require replication in larger, adequately powered trans-ethnic samples before firm conclusions about population-specific risk contributions can be drawn.

### 5.2 Mapping clinical variants to interaction domains

**Figure 8:**
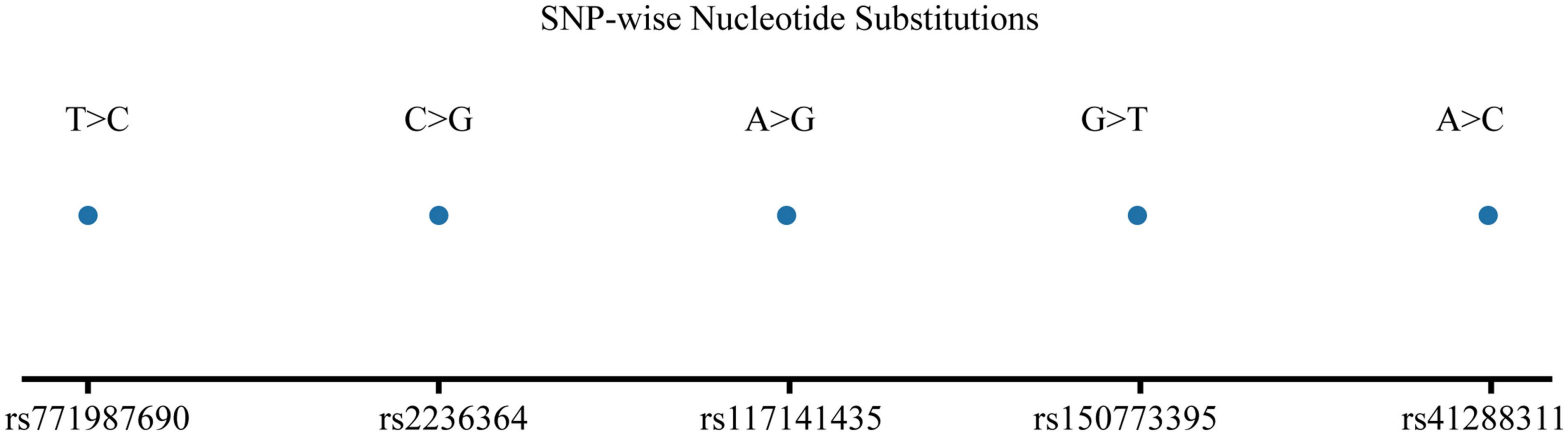
This graph shows the SNP ID of the variants found in the exon 8 of A-kinase anchor protein 353 11 and nucleotide substitution information.

In a Chinese bipolar disorder cohort, targeted sequencing of AKAP11 exon 8 failed to replicate the enrichment of ultra-rare protein-truncating variants reported in European populations, but identified five additional rare coding variants. Although no significant case–control association was observed, the population-specific distribution of these variants supports the need for larger trans-ethnic studies to clarify AKAP11-related risk mechanisms (Zhang et al., 2025), (Table 2, Figure 8).

**Table 2:** Rare variants identified in exon 8 of the AKAP11 gene in bipolar disorder patients.

Variant no.	Genomic position (hg38)	SNP ID	Nucleotide change	Variant type	Amino acid change
1	g.42300171 T > C	rs771987690	T → C	Synonymous	Asp475=
2	g.42300908 C > G	rs2236364	C → G	Missense	Ser721Cys
3	g.42301585 A > G	rs117141435	A → G	Missense	Ile947Val
4	g.42301991 G > T	rs150773395	G → T	Missense	Gly1082Val
5	g.42302154 A > C	rs41288311	A → C	Synonymous	Thr1136=

In addition to truncating variants, rare missense substitutions were identified within regions that encode known interaction domains, including a variant at rs2236364 that falls within a segment involved in PKA binding. Residues such as Thr1132 and the hydrophobic FLL motif (residues 1,162–1,176) have previously been implicated in GSK3β recruitment based on biochemical studies in cell lines (Whiting et al., 2015); however, whether the variants identified in this cohort alter these interactions in a functionally meaningful way has not been tested. Given the absence of a significant case–control association in this dataset, these observations should be regarded as preliminary, and functional interpretation awaits experimental validation in appropriate neuronal systems.

### 5.3 The “edgetic” mechanism: dysregulation vs. loss of function

Recent insights from structural systems biology suggest that many clinical AKAP11 variants act through an “edgetic” mechanism rather than a complete loss-of-function. In a classical loss-of-function model, a mutation eliminates the protein (the “node”) entirely from the interactome. In contrast, edgetic variants selectively disrupt specific protein–protein interactions (the “edges”) while leaving the scaffold largely intact and functional for other binding partners (Cui et al., 2023; Sahni et al., 2013).

In carriers of edgetic AKAP11 variants, the relationship between scaffold disruption and GSK3β activity is not straightforward. One proposed mechanism suggests that loss of AKAP11-mediated sequestration allows GSK3β to escape the localized inhibitory phosphorylation normally provided by anchored PKA, potentially increasing its activity within affected microdomains (Whiting et al., 2015). A separate line of evidence, however, reports that AKAP11 loss is associated with elevated cytosolic AKT1 activity, which in turn promotes inhibitory Ser9 phosphorylation of GSK3β, resulting in net reduction of its activity (Lee et al., 2024). These findings were obtained under different experimental conditions and may reflect distinct cellular contexts rather than a single unified mechanism. The net effect of AKAP11 disruption on GSK3β activity therefore remains unresolved, and conclusions about downstream signalling consequences should be drawn with caution until these discrepancies are addressed in controlled comparative studies.

**Figure 9:**
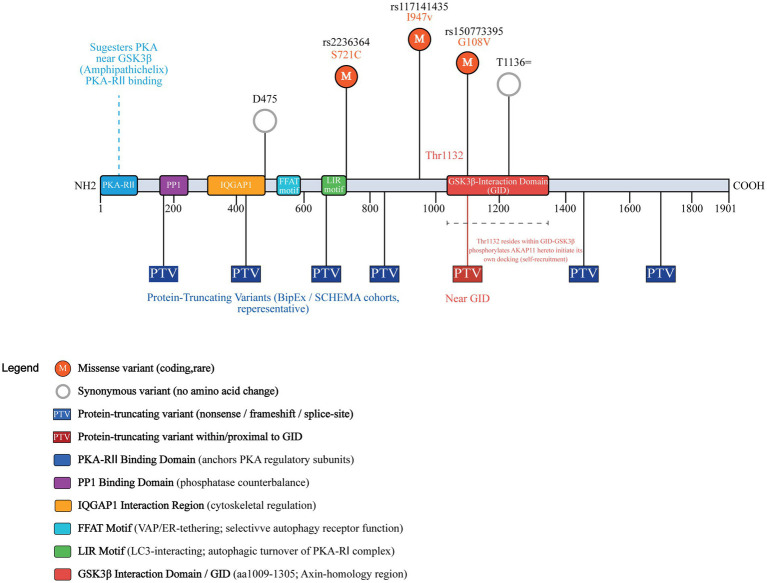
Stick map of A-kinase anchoring protein 11.

These observations are consistent with a model in which disruption of spatial signal fidelity may contribute to instability in kinase regulation, rather than a sustained reduction in enzymatic activity. This proposed “signalopathy” framework may offer a conceptual basis for understanding how such molecular alterations could be related to episodic features of bipolar disorder; however, direct evidence linking these mechanisms to mood-state transitions in carriers remains limited (Lee et al., 2024), (Figure 9).

### 5.4 Emerging proteostatic vulnerabilities

Recent studies have expanded the understanding of AKAP11 by identifying it as a selective autophagy receptor. Variants associated with bipolar disorder that affect the FFAT motif or the LC3-interacting region (LIR) have been shown in experimental systems to disrupt the autophagic turnover of the PKA-RI complex, leading to accumulation of regulatory subunits at the synapse. These results imply that the pathogenic effects of AKAP11 mutations may include improper clearance of signalling complexes and decreased spatial control of active kinases, which may contribute to the altered neuronal signalling and synaptic dysfunction seen in patients (Lee et al., 2024).

## 6 Functional consequences in neuronal cells

AKAP11-GSK3β disruption has been linked to a number of effects at three hierarchical levels that interact with each other within a cell (Zhang et al., 2026). These include synaptic transmission problems, alterations in the dendritic spine structure, and disruption of signaling integrity. Each of these will be described and explained in the next sections below.

### 6.1 Altered synaptic plasticity and transmission

Loss of AKAP11 or selective disruption of its interaction with GSK3β impairs basal synaptic transmission and plasticity. Electrophysiological recordings in AKAP11-deficient human induced neurons (iNeurons) and mouse models show reductions in miniature excitatory postsynaptic currents (mEPSCs), including decreases in both frequency and amplitude. This suggests a reduction in the number of functional synapses or altered postsynaptic AMPA receptor responsiveness (Lee et al., 2024).

These phenotypes appear to arise from compartment-specific activity disruption. Impaired AKAP11-mediated autophagy leads to the accumulation of PKA regulatory subunits (RIα/RIβ) at the synapse, which may sequester catalytic subunits and reduce dynamic kinase signalling during synaptic potentiation. Mislocalization of GSK3β can further disrupt NMDA receptor-dependent long-term depression (LTD), contributing to altered AMPA receptor internalization. Together, these mechanisms may compromise synaptic efficacy, although direct links to behavioral outcomes in humans remain to be fully established (Lee et al., 2024).

### 6.2 Disturbed dendritic spine dynamics

Structural synaptic plasticity, the ability of neurons to remodel their connections, is impaired when the AKAP11–GSK3β interaction is disrupted. Morphological analyses, including sparse labelling and electron microscopy of AKAP11-deficient neurons, show a reduction in dendritic spine density (Pallas-Bazarra et al., 2017; Aryal et al., 2023). AKAP11 deficiency has been associated with synaptic alterations that may preferentially impact dynamic spine populations, such as thin spines, which are known to be involved in synaptic plasticity and learning-related processes (Zhang et al., 2026; Carlisle and Kennedy, 2005; Bourne and Harris, 2007). Stability deficit in mushroom spines: Although “mushroom” spines (stable, mature connections) may initially persist, their long-term maintenance is compromised due to reduced formation of new spines (Ochs et al., 2015).

These structural changes are linked to GSK3β regulation of the cytoskeleton. Normally, spatial sequestration of active GSK3β by AKAP11 controls the phosphorylation of microtubule-associated proteins such as MAP1B and Tau at the spine base. Loss of AKAP11 scaffolding allows the kinase to phosphorylate these cytoskeletal components inappropriately, destabilizing the actin network within the spine head and leading to spine retraction (Medina et al., 2011).

### 6.3 Impaired neuronal signalling fidelity

At the core of the AKAP11-associated bipolar disorder phenotype lies a loss of signalling fidelity—defined as the ability of neurons to discriminate meaningful physiological signals from background activity. The AKAP11 scaffold normally maintains this precision by restricting kinase activation to discrete subcellular regions, allowing enzymes to respond only to localized, stimulus-dependent elevations in second messengers such as cAMP. Neuronal signalling becomes less spatially limited in the absence of this scaffold, which lowers the signal-to-noise balance (Lee et al., 2024). The neuron’s “signal-to-noise ratio” deteriorates in the absence of this scaffold. This failure is caused by two main mechanisms:

First, proteostatic stress: PKA regulatory subunit RI accumulates noticeably in the cytosol when AKAP11-dependent autophagic activities are compromised. Increased cytosolic PKA-RI modifies PKA complexes’ functional availability, hence buffering kinase activation and reducing neuronal reactivity to acute neuromodulatory inputs, such as dopamine spikes (Lee et al., 2024). Second, enzymatic mislocalization: loss of AKAP11-mediated sequestration allows GSK3β to phosphorylate substrates outside its normal signalling context. This aberrant kinase activity contributes to the engagement of maladaptive pathways, including stress-associated amygdalar hyperactivity and concurrent structural and functional decline of cognitive circuits within the prefrontal cortex, as observed in chronic stress models (Lee et al., 2024).

### 6.4 Alignment with bipolar disorder phenotypes

These cellular changes align with neurophysiological alterations observed in experimental model systems used to study bipolar disorder and schizophrenia, rather than directly reflecting patient-derived biotypes.

Apreclinical study was conducted to determine if mutations in GRIN2A and AKAP11 cause electroencephalography (EEG) abnormalities in mouse models for schizophrenia. EEGs in knockout mice showed elevated resting gamma frequency activity, decreased auditory steady-state responses at 40–50 Hz, and attenuated mismatch negativity responses to unpredicted auditory stimuli. Furthermore, sleep spindles were observed to be different, wherein mutants with decreased copies of the AKAP11 gene had fewer sleep spindles compared to those with a higher number of copies, while mutants with a defective Grin2a gene had more sleep spindles (Tondo et al., 2017).

Loss of AKAP11 leads to abnormal neuronal signalling, which manifests as behavioral instability in mice. In experimental tests, AKAP11-deficient mice show excessive activity when given stimulants, modeling mania-like behavior, and spend more time immobile in the forced swim test, modeling depressive-like behavior (Song et al., 2025). The disruption of the AKAP11-GSK3β interaction does not result in a complete loss of function. Instead, it creates a condition in which neurons are prone to signalling instability. Under normal, low-demand conditions, the neurons continue to operate, but during periods of heightened activity, their signalling capacity is compromised. This provides a potential biological framework for understanding the switch-like, episodic patterns observed in bipolar disorder (Table 3).

**Table 3:** This table summarizes key synaptic and circuit-level molecular abnormalities and links those to specific neuropsychiatric symptoms, emphasizing how disturbed signalling, structure, and proteostasis converge on cognitive and behavioral dysfunction.

Functional defect	Molecular cause	Clinical phenotype correlation	Source
Reduced mEPSC frequency	AMPA receptor internalization/loss of synapses	Cognitive blunting; anhedonia	(Lee et al., 2024)
Thin spine erosion	Cytoskeletal instability (Tau/MAP1B)	Impaired learning; chronic mood cycling	(Medina et al., 2011)
RIα/RIβ accumulation	Autophagy receptor failure	Proteostatic stress; signal damping	(Cui et al., 2023)
Increased gamma power	PV+ interneuron/excitatory-inhibitory (E/I) imbalance	Disorganized thinking; psychosis	(Medina et al., 2011)

## 7 Role in bipolar disorder pathophysiology

Bipolar disorder is characterized by alternating periods of high and low energy, which indicate episodic mood changes (Farhangdoost et al., 2025). These changes can be explained molecularly by the interaction between AKAP11 and GSK3β, which moves us away from static chemical imbalance models and toward a viewpoint focused on dynamic instability in neural signalling (Palmer et al., 2022; Lee et al., 2024).

### 7.1 Stronger association with bipolar disorder

Although there is genetic overlap across psychotic disorders, the association with AKAP11 is notably stronger and more specific to bipolar disorder. Large-scale exome sequencing studies show that individuals carrying protein-truncating variants in AKAP11 have approximately a seven-fold increased risk of developing BD, one of the largest effect sizes reported in psychiatric genetics. This elevated risk is particularly concentrated in patients with psychosis-related forms of BD, indicating that disruption of the AKAP11 scaffold interferes with the neural circuits underlying higher-order cognitive and emotional processing. Unlike other high-impact risk genes that also increase susceptibility to intellectual disability, AKAP11 variants appear to selectively impair the signalling mechanisms essential for mood regulation, providing a more targeted genetic model for investigating cyclic bipolar disorder (Palmer et al., 2022).

### 7.2 Episodic signalling instability vs. chronic defect

Traditional models in biological psychiatry often view mental disorders as persistent and static deficits. The AKAP11 scaffold, on the other hand, suggests that those with detrimental mutations have episodic instability in neural signalling. By acting as a molecular “dam,” AKAP11 keeps PKA and GSK3β activity within safe, homeostatic bounds. The neurons that have lower expression levels for AKAP11 would function normally under normal conditions. But there would be peaks in levels of the signalling molecule, that is, cAMP or dopamine, when demands are higher, for example, under stress conditions or during interrupted sleeping times.

These surges are no longer regulated without the presence of the spatial regulation of AKAP11, which means that the enzymes are allowed to phosphorylate targets that they are not supposed to phosphorylate in the synapse. The uncontrolled phosphorylation means that there is uncontrolled signalling, which offers a biological model for the episodic nature of bipolar disorder. A patient with bipolar disorder may be asymptomatic for a period of several years, but the complex signalling pathway goes haywire with certain triggers, leading to sudden shifts to mania and depressive episodes (Palmer et al., 2022).

### 7.3 Abnormal GSK3β signalling cycles

Impaired AKAP11 functionality results in the dysregulation of feedback mechanisms, which modulate GSK3β activity. In a normal neuron, the activated GSK3β promotes its own translocation to the AKAP11 scaffold complex by a phosphorylative event at residue Thr1132, where the enzymatic activity of PKA is repressed by its presence in complex with protein kinase inhibitor, PKI:

1. Aberrant activation: Without the scaffold’s spatial constraint, GSK3β is exposed to activating signals outside of its typical microdomain (Lee et al., 2024).
2. Impaired inhibition: Because anchored PKA is unable to effectively phosphorylate Ser9 on GSK3β, the kinase remains active for extended periods of time (Lee et al., 2024).

### 7.4 Explaining mood switching behavior

The interaction between AKAP11 and GSK3β provides a mechanistic framework for the rapid switching between manic and depressive episodes in bipolar disorder. In manic states, dopamine-driven cAMP–PKA signalling becomes excessively amplified, while in depressive phases, signalling is attenuated, synapses are destabilized, and structural plasticity is lost. Disruption of AKAP11 uncouples this finely tuned regulatory system, resulting in abnormal accumulation of PKA regulatory subunit I (PKA-RI) and compression of the neuron’s effective signalling range (Song et al., 2025).

#### 7.4.1 The manic switch

Dopaminergic signaling can modulate intracellular cascades that include PKA activity and downstream targets such as GSK3β (Beaulieu et al., 2007). AKAP11 functions as a scaffolding protein that helps organize and localize PKA signaling complexes, thereby contributing to the spatial and temporal regulation of kinase activity within neurons (Whiting et al., 2015). Disruption of AKAP11 may therefore alter the organization and efficiency of these signaling complexes, potentially affecting the balance of kinase signaling pathways. Such alterations have been implicated in the broader neurobiology of mood regulation (Zhang et al., 2026) and are consistent with mechanisms that may contribute to mood instability and manic-like phenotypes, although the relationship is likely indirect and influenced by multiple interacting biological and environmental factors (Lee et al., 2024).

#### 7.4.2 The depressive switch

This perpetual instability in signalling translates into proteostatic stress due to the constant turnover of proteins, leading to synaptic exhaustion. The selective loss of thin, plastic dendritic spines compromises circuitry underlying learning deficits, causing immobility, anhedonia, and cognitive slowing seen with bipolar depression (Song et al., 2025).

When these elements combine, there is an inability to sustain a euthymic homeostatic set-point because the brain cycles between two pathological extremes, resulting in a metastable neural state. This model is validated by the success of lithium therapy, because pharmacologic correction of a kinase node can restore equilibrium to signalling systems that are perturbed by AKAP11 because of its GSK3β inhibition.

## 8 Therapeutic implications

The recognition of AKAP11 as an important genetic contributor for bipolar disorder reveals major future advancements in the understanding, diagnosis and treatment of psychiatric care. Rather than justifying the standard treatment approach that focuses on pharmacologically based symptomatic relief, there is now scope to explore targeted therapy based on clearly established molecular pathways. The identification of AKAP11-GSK3β signalling microdomain as the crucial susceptibility site redefines bipolar disorder as a context-dependent signalling dysfunction as opposed to a diffuse neurochemical dysregulation. The clear understanding of mechanistic pathways forms a rational basis for treatment approaches aiming at regulating intercellular signalling to treat the underlying cause of episodic instability rather than solely focusing on symptomatic relief.

### 8.1 Lithium as a functional proxy for the scaffold

Emerging preclinical evidence suggests that the therapeutic effects of lithium may partially intersect with the AKAP11–GSK3β signalling axis. A 2025 study reported that prolonged lithium treatment was associated with partial restoration of aberrant enhancer activity and gene expression patterns in human neurons carrying heterozygous AKAP11 loss-of-function mutations, bringing their transcriptional profile closer to that of wild-type neurons (Farhangdoost et al., 2025). At the mechanistic level, lithium promotes inhibitory phosphorylation of GSK3β at Ser9 (Post et al., 2025), which may partially compensate for the loss of scaffold-mediated spatial regulation. These findings are conceptually consistent with the hypothesis that lithium’s therapeutic action could be relevant in AKAP11 variant carriers; however, this remains speculative and has not been tested in prospective clinical studies.

Regarding lithium response in AKAP11 variant carriers specifically, available data are extremely limited. The BipEx study reported lithium response information for only 11 carriers of AKAP11 protein-truncating variants, of whom seven reported a favourable response (Palmer et al., 2022). The authors of that study explicitly cautioned that this sample size was insufficient to draw any meaningful conclusions. No adequately powered prospective study has examined lithium response stratified by AKAP11 genotype, and the suggestion of a genotype-specific response advantage must therefore be regarded as a hypothesis requiring dedicated investigation rather than an established clinical observation.

### 8.2 Moving beyond indirect enzymatic modulation

Current pharmacological approaches to mood stabilization rely largely on indirect modulation of broad enzymatic targets. Because GSK3β and PKA are essential for cellular processes throughout the body, systemic inhibition of these kinases carries inherent risks of off-target effects in non-neuronal tissues. From a theoretical standpoint, the AKAP11–GSK3β protein–protein interaction interface represents an interesting candidate for more spatially selective intervention, given that disruption of this specific interaction—rather than global kinase inhibition—appears to drive signalling dysregulation in experimental models. In principle, small molecules or peptide-based compounds designed to stabilize or restore this interaction could offer a more targeted approach than pan-GSK3β inhibitors. However, no such compounds have been developed or tested to date. Critical obstacles remain, including the absence of a high-resolution co-crystal structure of the AKAP11–GSK3β complex, the intrinsically disordered nature of large regions of AKAP11, and the lack of any *in vivo* pharmacological validation. These constraints mean that therapeutic targeting of this interface, while conceptually motivated, remains a distant and unproven prospect.

### 8.3 Potential for precision-based therapy

The identification of AKAP11 as a high-impact risk gene has prompted theoretical interest in precision-based approaches that could 1 day stratify patients by molecular mechanism rather than clinical phenotype alone. Several hypothetical strategies have been proposed in the literature, including small molecules aimed at stabilizing disrupted AKAP11–GSK3β binding in carriers of interaction-domain variants, agents that might enhance autophagic clearance of accumulated PKA-RI complexes, and ion channel modulators that could address downstream hyperexcitability (Farhangdoost et al., 2025; Goodchild et al., 2024). These proposals are intellectually coherent given what is currently known about AKAP11 biology, but it is important to emphasize that none of them have progressed beyond conceptual discussion. No candidate compound has been identified, no target engagement study has been conducted in relevant neuronal models, and no clinical biomarker exists to stratify AKAP11 variant carriers for treatment selection. The path from a genetically defined molecular mechanism to a validated therapeutic strategy requires extensive preclinical and clinical development that has not yet begun for this target. The interaction domain between AKAP11 and GSK3β, therefore, represents a hypothesis-generating framework for future drug discovery rather than an actionable therapeutic target in its current state.

## 9 Limitations

Even while the discovery of AKAP11 as a major risk gene has yielded profound molecular insights, there are still substantial obstacles that impede the application of this understanding in clinical settings. These challenges include methodological, structural, and practical constraints; thus, the results of this study should be regarded with caution.

### 9.1 Scarcity of high-resolution structural data

The lack of a complete, high-resolution crystal structure of AKAP11 is a significant obstacle to researching the AKAP11-GSK3β interaction. With 1,901 amino acids, AKAP11 is a fairly big protein with several intrinsically disordered areas that make structural characterization challenging. Researchers are presently employing advanced computational techniques like AlphaFold3 to anticipate the structure of the GSK3β Interaction Domain (GID) and its position within the protein complex. Although these predictions are helpful, they lack the atomic-level information required to create tiny compounds that precisely target the interaction between proteins. Furthermore, the AKAP11-GSK3β-PKA complex lacks co-crystal structures, which are essential for comprehending the spatial organization of this “signal-regulating” scaffold.

### 9.2 Reliance on indirect interaction methodologies

The connection between AKAP11 and GSK3β is largely supported by indirect or static ways. Proximity labeling (BioID), co-immunoprecipitation, and yeast two-hybrid screening are good methods for finding possible binding partners, but they do not demonstrate how these associations alter in real time during signalling events. The majority of research has been conducted in post-mortem brain tissue or overexpressed systems like HEK293 cells, which may not fully represent how these proteins function normally in living neurons. Currently, there are no biosensors available to monitor the AKAP11-GSK3β complex in real time, for example, during mood fluctuations or stress responses, leaving a gap in understanding the dynamics of this interaction in living brains.

### 9.3 Insufficiency of in vivo validation

Animal studies, especially those using AKAP11 knockout or heterozygous mice, have offered important insights into neurophysiology, but translating these findings to humans has limitations. Germline knockouts, which can cause developmental compensations that do not happen in adults with bipolar disorder, are used in the majority of research. Furthermore, the intricate polygenic landscape of real bipolar disorder, where AKAP11 mutations interact with numerous other small-effect risk genes, is not captured by these models. Additionally, there is a lack of long-term validation: whereas AKAP 11-deficient mice exhibit behaviors like sadness or mania, it is unclear if these behaviors truly represent the cyclical and self-limiting mood episodes observed in people over the course of a lifetime.

## 10 Future directions

It will be necessary to combine state-of-the-art structural biology, thorough multi-omics investigations, and sophisticated predictive machine learning to bridge the gap between AKAP11’s molecular signalling abnormalities and the clinical presentations of bipolar disorder. In order to create interventions that specifically target these fundamental systems, future research should concentrate on four main areas.

### 10.1 High-resolution structural mapping of interaction domains

The major challenge for understanding the full architecture of this giant scaffold protein with 1901 amino acids is presented. In this regard, AI-driven structural biology approaches must be combined with emerging approaches in quantum computing (Doga et al., 2024) to enable such understanding. AlphaFold 3 is capable of predicting the joint structure of protein complexes, including AKAP11 bound to GSK3β and PKA, which will provide more information on the spatial organization of the “signalostat.” Quantum computing67 may accelerate this process by efficiently exploring the enormous conformational space of such large complexes and spotting energetically favorable states that might reveal subtle interactions difficult to observe classically.

### 10.2 Neuron-type and compartment-specific studies

A recent multi-omics analysis reveals that a loss-of-function mutation in AKAP11 causes dysregulation of PKA signaling throughout the brain, including the cortex, striatum, hippocampus, and thalamus, along with extensive changes in transcriptome and synaptic gene expression in the striatum (Song et al., 2025). Future research should focus on specific cell types of neurons, primarily parvalbumin-positive (PV+) GABAergic interneurons, which are vital to the generation of gamma oscillations that are often impaired in psychosis-related circumstances (Kuki et al., 2015).

Moreover, since AKAP11 is an autophagy receptor for the PKA-RI complex, it is also important to consider the differences in signalling activity that exist within the neurites and within the cellular body (soma). The specific mechanisms that occur within the synaptic areas that cause weakening can also be explained by understanding the PKA-RI complex and other signalling molecules that accumulate in cellular synapses rather than in the cytosol.

### 10.3 Integration of molecular and clinical data streams

The next development in psychiatry is the integration of digital tools with molecular biology. By connecting a person’s genetic profile, such as AKAP11 interaction-disrupting variations, with real-time mood monitoring, machine learning models, such as TabPFN (Hollmann et al., 2025) and Gaussian processes, can assist in predicting individual mood patterns. The relationship between certain mutations and clinical outcomes such as lithium responsiveness, cognitive alterations, or metabolic consequences may be shown using this “population edgetics” method. We can advance toward really individualized, mechanism-based mental health therapy by combining information from behavior, brain activity, and protein structures.

## 11 Conclusion

According to this review, AKAP11 is a key regulator of the brain’s spatial GSK3β signalling. AKAP11 mutations have been genetically linked to psychosis and bipolar disorder. AKAP11 regulates the location and timing of GSK3β activity, according to structural and interaction investigations. Kinase activity becomes mislocalized when this scaffold is disrupted. This results in reduced plasticity and synaptic instability. Proteostatic stress, spine loss, and decreased mEPSCs are demonstrated by functional data. Excitatory-inhibitory imbalance and aberrant gamma oscillations are examples of circuit-level impacts. These alterations are consistent with mood and cognitive impairment. Crucially, disease results from a lack of spatial control rather than a total loss of kinase activity. This explains why global GSK3β inhibitors have little selectivity. An induced-fit interaction at the AKAP11 GSK3β-binding domain is supported by AlphaFold-based modelling. A specific therapeutic target is represented by this interface. Model systems and insufficient structural resolution limit the available evidence. Future research must connect particular variations to functional results. A precise and biologically sound therapeutic approach is to target scaffold-mediated signalling.
